# The moderating effect of body mass index on the association between lung function and physical fitness among adolescents

**DOI:** 10.3389/fpubh.2026.1867007

**Published:** 2026-08-07

**Authors:** Yi Fang, Juhao Hou, Shilin Wen

**Affiliations:** Institute of Physical Education and Training, Capital University of Physical Education and Sports, Beijing, China

**Keywords:** adolescents, body mass index, physical education, physical fitness, school sports, vital capacity

## Abstract

**Objective:**

To investigate the moderating effect of body mass index (BMI) on the association between lung function and physical performance in middle school students, providing quantitative empirical evidence for the implementation of precision physical education.

**Methods:**

A total of 493 eighth-grade students from a middle school in Beijing were recruited for this study. BMI, vital capacity, and various physical fitness indicators were assessed, and the participants were categorized into four groups: normal weight, overweight, obese, and underweight. Spearman rank correlation analysis with Bonferroni correction was employed to evaluate the relationship between lung function and physical fitness across different BMI groups. Following mean-centering of the variables, hierarchical regression models combined with Bootstrap resampling were constructed to examine the interaction and moderating effects.

**Results:**

The association between lung function and physical fitness exhibited significant BMI-stratified heterogeneity. In the normal-weight group, vital capacity correlated with the standing long jump (boys: *r* = 0.329, *p* < 0.001; girls: *r* = 0.252, *p* < 0.001) and 50-m sprint (boys: *r* = −0.257, *p* < 0.001; girls: *r* = −0.230, *p* < 0.01), as well as the boys’ 1,000-m run (*r* = −0.192, *p* < 0.01). In overweight boys, it negatively correlated with the 50-m sprint (*r* = −0.557, *p* < 0.05) and 1,000-m run (*r* = −0.698, *p* < 0.001). Global quadratic interaction terms lacked overall significance (*p* > 0.05). However, the boys’ quadratic BMI_c^2^ term showed a non-linear trend in the 50-m sprint [95% CI (0.002, 0.025)] and significance in the 1,000-m run (*β* = 0.340, *p* = 0.002). Conversely, girls’ running events only showed a BMI_c main effect (*β* = 0.391, *p* < 0.001); their standing long jump maintained a significant Vital Capacity_c main effect (*β* = 0.221, *p* = 0.014) and a linear interaction effect [Vital Capacity_c × BMI_c: 95% CI (0.019, 0.434)].

**Conclusion:**

The moderating effect of BMI on adolescent vital capacity and athletic performance demonstrates significant stratified heterogeneity. The translation of vital capacity into physical fitness diverges once specific BMI thresholds are surpassed. Accordingly, physical education should abandon ‘one-size-fits-all’ approaches in favor of precision interventions integrating individual physiological profiles.

## 1 Introduction

In recent years, the “Four Minors” health dilemmas have become increasingly pronounced among the Chinese youth population. According to data from the 8th National Student Physical Fitness and Health Survey, the detection rate of overweight and obesity among primary and secondary school students in China reached 19.1% in 2019, representing a 3.2% increase compared to 2014; concurrently, although the detection rate of underweight has declined overall, it remains stubbornly above 8% in certain economically underdeveloped regions (1). Meanwhile, although absolute vital capacity levels have demonstrated a rebounding trajectory after a prior decline, the vital capacity-to-weight index exhibits significant interval heterogeneity across students of different body types, with prominent discrepancies persisting between urban and rural areas as well as across distinct somatotypes (2). To address this issue, the state has issued a series of guiding policies. However, limited by practical factors such as venues, resources, and teaching staff, translating policy requirements into effective teaching practices still urgently requires a systematic answer at the empirical level. Crucially, the continuous surge in overweight and obesity rates among children and adolescents serves as one of the primary catalysts constraining the improvement magnitude of their relative lung function. The root causes of these predicaments are multifaceted, fundamentally epitomizing a systemic absence of collaborative synergy among families, schools, and society (3). To overcome these systemic bottlenecks and translate national policy mandates into tangible pedagogical practices, a paradigm shift toward precision physical education is urgently needed. Within the scope of this study, precision physical education is defined as an educational process that leverages modern information technology to scientifically collect and analyze data concerning students’ physical fitness, motor skills, and psychological states. This approach enables the dynamic adaptation of instructional objectives, curricular content, teaching methodologies, and evaluation frameworks, thereby satisfying the differentiated athletic demands of diverse individuals while elevating the efficiency and quality of physical education (4).

Within the academic landscape of adolescent physical health research, body mass index (BMI), pulmonary function, and physical performance constitute a central area of inquiry (5). Vital capacity, serving as a static volumetric indicator of pulmonary ventilation, shares a direct physiological linkage with physical exercise capacity. It determines or influences the volume of oxygen an individual can inspire per unit of time, thereby modulating aerobic metabolic capacity and subsequent athletic performance. Regarding BMI, a widely utilized metric for body composition, previous studies have established a non-linear correlation between BMI and vital capacity levels. For instance, a quantile regression analysis involving 10,800 primary school students in ethnic minority regions of Sichuan demonstrated that the association between vital capacity and BMI exhibits a robust positive correlation within the middle distribution range, whereas this relationship is significantly attenuated at the extreme BMI quantiles (6). Similarly, research focusing on pediatric asthma cohorts has identified a critical threshold effect between the BMI Z-score and pulmonary function: once the BMI Z-score exceeds 3.945, each 1-unit increment is associated with a 9.7% decline in FEV₁ and a 12.2% reduction in PEF, marking an inversion of the pulmonary function-weight relationship from positive to negative (7). Collectively, these studies unveil a crucial paradigm: the impact of BMI on pulmonary function does not follow a monotonic linear trajectory, but rather manifests as a characteristic “inverted U-shaped” curve.

The multidimensional impact of body mass index (BMI) on physical performance has also garnered widespread attention within the academic community. Among children and adolescents, the underlying mechanisms linking obesity to diminished physical fitness have been elucidated in finer detail (8). A study focusing on obese children and adolescents aged 7 to 18 revealed that while metabolic syndrome significantly impairs functional exercise capacity, the negative impact of obesity indices on exercise tolerance far outweighs that of the metabolic syndrome itself (9). Furthermore, increased abdominal circumference and elevated fasting blood glucose emerged as key independent predictors of diminished pulmonary function and exercise tolerance. In addition, a study involving Zhuang primary school students aged 7 to 12 in Guangxi demonstrated that BMI, waist circumference, and body shape index can all significantly predict maximal oxygen uptake levels. Notably, the negative predictive effect of body composition on cardiorespiratory endurance exhibits a high degree of consistency across diverse geographical regions and ethnic groups (10).

While the aforementioned studies unveil a non-linear association between BMI and vital capacity among children and adolescents, previous literature has rarely integrated “vital capacity,” “physical fitness,” and “BMI” simultaneously into a unified analytical paradigm. Therefore, this study aims to explore the moderating role of BMI in the relationship between adolescent lung function and physical fitness by constructing a non-linear moderating effect model. Rather than merely focusing on the simple correlation between the two, this research places particular emphasis on elucidating whether the magnitude or direction of the association between lung function and physical performance undergoes significant alterations across distinct BMI sub-groups, thereby providing a robust scientific basis for differentiated physical education interventions.

## 2 Methods

### 2.1 Measurement methods

#### 2.1.1 Participants

Eighth-grade students from a middle school in Beijing were selected as the participants for this study, and the tests were conducted in March 2026. Initially, 592 students were recruited; after excluding those with incomplete data, a valid sample of 493 participants remained, comprising 267 boys and 226 girls. This study strictly adhered to the specific cut-off points for eighth-grade boys and girls outlined in the *National Student Physical Fitness and Health Standard (2014 Revision)* (11)issued by the Ministry of Education to categorize the participants’ body mass index (BMI, kg/m^2^). The specific classification criteria are as follows: boys were categorized as underweight (≤15.6), normal weight (15.7–22.5), overweight (22.6–25.2), and obese (≥25.3); girls were categorized as underweight (≤15.2), normal weight (15.3–22.2), overweight (22.3–24.8), and obese (≥24.9). Based on the aforementioned criteria, the final sample composition was: 404 in the normal-weight group, 34 in the overweight group, 18 in the obese group, and 37 in the underweight group. All students were in good health, free from severe cardiopulmonary diseases and contraindications to exercise, and capable of completing the physical fitness tests normally. Prior to testing, the objectives and procedures were explained in detail to the students and their parents, and informed consent was obtained.

#### 2.1.2 Test indicators and instruments

The selection of indicators was based on the regulations for student physical fitness testing stipulated in the National Student Physical Fitness and Health Standard (2014 Revision) (11). The specific test indicators and corresponding instruments are determined as follows:

① Anthropometric indicators: height (cm) and weight (kg). Measurements were taken using an electronic height and weight scale (Model: HGM-300; Zhengzhou Hengxing Electronic Co., Ltd., China). The body mass index was calculated as: BMI = weight (kg) / height^2^ (m^2^).
② Pulmonary function indicator: vital capacity (ml). A smart electronic spirometer (Model: Taimeiquan Vital Capacity Tester; Beijing Taimeiquan Technology Co., Ltd., China) was used for measurement. To ensure scientific validity, students were required to fast and refrain from vigorous exercise for 2 h prior to the test, and to familiarize themselves with the breathing protocol. During the test, participants assumed a standing position, took a maximal deep inspiration, and then exhaled as forcefully and deeply as possible into a disposable mouthpiece. Each participant performed the test twice, with a resting interval of at least 30 s between trials. Each exhalation was required to be a continuous, explosive airflow without mid-way air leakage. The system automatically extracted and recorded the maximum value from the two valid trials.
③ Physical fitness indicators: 50-m sprint (s), standing long jump (cm), 1,000-m run for boys (s), 800-m run for girls (s), pull-ups for boys (reps), and one-minute sit-ups for girls (reps). The testing instruments for the above indicators correspondingly included a stopwatch, a measuring tape with a standing long jump mat, a standard horizontal bar, and an electronic sit-up tester. Although the sit-and-reach test was conducted using a sit-and-reach box in accordance with national standards, it was excluded from the subsequent correlation and regression analyses. This decision was made because flexibility lacks a direct physiological and biomechanical association with pulmonary function and cardiorespiratory endurance, thereby ensuring the concentrated focus of the study’s core hypothesis.

#### 2.1.3 Test procedure

All tests were uniformly conducted on the school’s standard outdoor synthetic athletic track during regular morning physical education classes and morning exercise sessions. On the day of testing, students were required to wear sportswear and athletic shoes, and were instructed to avoid taking the tests on an empty stomach or immediately after a heavy meal. The testing methodologies and frequencies for each specific item were as follows: ① Height and weight: Tested once. Participants stood barefoot in an upright position, and the readings were recorded. ② Vital capacity: Tested twice, and the maximum value was recorded. A rest interval of at least 30 s was strictly enforced between trials. ③ Sit-and-reach: Tested twice, and the best score was recorded. A 15-s rest interval was provided between trials. ④ Standing long jump: Tested three times, and the best score was recorded. A 30-s rest interval was provided between jumps. ⑤ 50-m sprint: Tested twice. Students ran in groups of 2 to 4 utilizing a standing start, timed with a stopwatch. ⑥ Pull-ups (boys): Tested once. Utilizing a pronated (overhand) grip on the horizontal bar, one valid repetition was counted when the chin cleared the bar. The total number of completed repetitions was recorded. ⑦ Sit-ups (girls): Tested once. Timed for exactly 1 min, the total number of correctly performed repetitions was recorded. ⑧ 1,000-m / 800-m run: Tested once. Students ran in groups of 8 to 15 utilizing a standing start, timed with a stopwatch to the nearest hundredth of a second.

### 2.2 Statistical methods

All data were processed and analyzed using the Statistical Package for the Social Sciences (SPSS, version 27.0, IBM Corp., Armonk, NY, United States). Initially, descriptive statistics were conducted for all test indicators, with continuous variables expressed as mean ± standard deviation. Prior to the correlation analysis, the Shapiro–Wilk test was employed to assess the normality of the data across all groups. Given that the sample data of the normal-weight BMI group did not fully satisfy the assumption of normal distribution, this study utilized the more robust Spearman rank correlation analysis to explore the correlations between vital capacity and various physical fitness test scores among boys and girls across different BMI sub-groups, thereby ensuring the uniformity of statistical criteria and the reliability of the results. Meanwhile, to strictly control the false positive rate arising from multiple comparisons, the Bonferroni correction (*k* = 4) was introduced into the bivariate correlation analysis. Since vital capacity was tested for correlation with four physical fitness indicators within each gender and BMI subgroup, the nominal *p*-value threshold for statistical significance was adjusted to 0.0125 to maintain a family-wise error rate of 0.05.

To formally examine the moderating role of BMI in the relationship between vital capacity and physical fitness, hierarchical linear regression models were constructed. To avert multicollinearity issues, prior to constructing the interaction and quadratic terms, the continuous independent variables—BMI and vital capacity—were subjected to mean-centering, yielding variables denoted as Vital Capacity_c and BMI_c. Subsequently, the centered main effect variables, the quadratic term of BMI_c (BMI_c^2^), and the interaction terms (Vital Capacity_c * BMI_c, Vital Capacity_c * BMI_c^2^) were sequentially entered into the models. Furthermore, the Bootstrap method (with 1,000 resamples) was utilized to estimate the robust 95% confidence intervals (95% CIs) for the interaction terms (12). Given that the empirical distribution of regression coefficients generated by Bootstrap resampling might be skewed, the approximate *p*-values calculated based on Bootstrap standard errors and the 95% CIs constructed via the percentile method could, in rare instances, yield divergent inferences. Consequently, this study adopted whether the Bootstrap 95% percentile confidence interval encompassed zero as the ultimate criterion for determining the statistical significance of the regression coefficients; if the confidence interval did not encompass zero, the effect was deemed significant at the *α* = 0.05 level. Concurrently, the approximate *p*-values calculated based on Bootstrap standard errors were reported for readers’ reference. All statistical tests were two-sided, with the significance level set at *p* < 0.05. Additionally, multicollinearity diagnostics revealed that the Variance Inflation Factor (VIF) for all predictor variables was well below 3.0, indicating the absence of severe multicollinearity issues; residual analysis also confirmed that the assumptions of normality and homoscedasticity were adequately met (13, 14).

## 3 Result

### 3.1 Descriptive statistical results

Table 1 presents the baseline demographic characteristics of the participants. All subjects selected for this study were eighth-grade students with a mean age of approximately 14 years. Table 2 delineates the descriptive statistical characteristics of various physical fitness indicators stratified by gender and BMI categories. For the male cohort, physical performance exhibited a pronounced trend of morphological differentiation; the normal-weight BMI group demonstrated optimal proficiency across the 50-m sprint, 1,000-m run, standing long jump, and pull-up tests, whereas the obese subgroup consistently lagged behind all other groups across all fitness domains. Crucially, however, the mean absolute vital capacity of overweight and obese boys surpassed that of the other groups. Conversely, the distribution of physical advantages within the female cohort demonstrated distinct item-specificity: the underweight group recorded the shortest average times in both the 50-m sprint and the 800-m run, the overweight group achieved the most prominent scores in the sit-up test, and the highest mean absolute vital capacity was concurrently captured within the obese subgroup.

**Table 1:** Baseline demographic characteristics of students.

Gender	*N*	Height (cm)	Weight (kg)	BMI (kg/m^2^)
Male	267	167.51 ± 8.25	53.89 ± 11.19	19.08 ± 2.97
Female	226	163.42 ± 5.29	50.07 ± 7.97	18.75 ± 2.74

**Table 2:** Descriptive statistics of physical fitness test results for male and female students.

Gender	BMI category	*n*	50-m sprint (s)	Standing long jump (cm)	1,000-m/800-m run (s)	Pull-ups/Sit-ups (reps)	Vital capacity (mL)
Male	Underweight	21	8.50 ± 0.79	193.63 ± 19.68	276.17 ± 44.88	3.75 ± 7.73	3129.71 ± 777.35
	Normal	216	8.13 ± 0.73	200.74 ± 20.21	250.36 ± 25.90	4.54 ± 5.91	3506.22 ± 666.88
	Overweight	20	8.26 ± 0.88	194.80 ± 21.58	270.25 ± 37.65	3.15 ± 4.80	4119.85 ± 639.21
	Obese	10	9.02 ± 0.89	177.20 ± 21.31	303.80 ± 32.55	1.50 ± 4.09	4066.70 ± 808.26
Female	Underweight	16	8.68 ± 0.59	176.56 ± 13.56	218.63 ± 20.09	47.56 ± 11.15	2657.56 ± 278.10
	Normal	188	8.90 ± 0.60	171.79 ± 15.39	226.63 ± 14.20	46.57 ± 7.61	3019.87 ± 521.47
	Overweight	14	8.86 ± 0.41	172.79 ± 11.87	229.71 ± 14.89	50.86 ± 7.03	2853.07 ± 230.36
	Obese	8	9.29 ± 0.49	176.50 ± 10.60	258.38 ± 17.75	47.88 ± 7.34	3059.13 ± 248.68

The 1,000-meter run and pull-ups are the test items for male students, while the 800-meter run and sit-ups are for female students.

### 3.2 Correlation analysis between vital capacity and physical performance across different BMI groups by gender

To preliminarily explore the relationship between vital capacity and physical performance, this study conducted Spearman rank correlation analyses stratified by gender and BMI categories, employing the Bonferroni correction for rigorous adjustment. Concurrently, in response to statistical norms advocating against an over-reliance on *p*-values, this section emphatically incorporates the correlation coefficient (r) to intuitively present the effect sizes of the associations (detailed results are presented in Table 3).

**Table 3:** Correlation analysis of vital capacity and various physical fitness indicators among male and female students across different BMI groups.

Gender	BMI category	*N*	50-m Sprint		Standing Long Jump		800-m (F) / 1,000-m (M) Run		Sit-ups (F) / Pull-ups (M)	
			r	95% CI	r	95% CI	r	95% CI	r	95% CI
Male	Underweight	21	−0.317	[−0.646, 0.112]	0.437	[0.028, 0.721]	−0.392	[−0.693, 0.026]	0.352	[−0.072, 0.669]
	Normal	216	−0.257***	[−0.382, −0.123]	0.329***	[0.199, 0.447]	−0.192**	[−0.321, −0.055]	0.163	[0.025, 0.295]
	Overweight	20	−0.557*	[−0.807, −0.138]	0.426	[−0.034, 0.737]	−0.698***	[−0.875, −0.357]	0.334	[−0.141, 0.684]
	Obese	10	−0.315	[−0.796, 0.411]	0.372	[−0.356, 0.819]	−0.236	[−0.763, 0.479]	0.156	[−0.541, 0.726]
Female	Underweight	16	0.096	[−0.433, 0.576]	−0.254	[−0.675, 0.291]	0.3	[−0.245, 0.701]	−0.356	[−0.732, 0.185]
	Normal	188	−0.230**	[−0.365, −0.085]	0.252***	[0.108, 0.385]	−0.11	[−0.254, 0.038]	0.113	[−0.035, 0.256]
	Overweight	14	−0.207	[−0.674, 0.378]	0.559	[0.024, 0.846]	−0.502	[−0.821, 0.057]	−0.06	[−0.584, 0.500]
	Obese	8	−0.503	[−0.897, 0.336]	−0.263	[−0.825, 0.560]	−0.286	[−0.833, 0.543]	−0.238	[−0.816, 0.578]

*p < 0.05, **p < 0.01, ***p < 0.001 based on Bonferroni correction. The bold font indicates that the corresponding indicator is statistically significant.

Among boys in the normal-weight group, vital capacity exhibited a significant positive correlation with the standing long jump (*r* = 0.329) and significant negative correlations with both the 50-m sprint (*r* = −0.257) and the 1,000-m run (*r* = −0.192), whereas its association with pull-ups did not reach statistical significance (*r* = 0.163). Within the overweight boys’ subgroup, the negative associations between vital capacity and running performances were substantially more pronounced; vital capacity was significantly negatively correlated with the 50-m sprint (*r* = −0.557) and highly significantly negatively correlated with the 1,000-m run (*r* = −0.698), but failed to achieve significant correlations with either the standing long jump (*r* = 0.426) or pull-ups (*r* = 0.334). In contrast, for boys in the underweight and obese subgroups, the correlation coefficients between vital capacity and all physical fitness test items did not attain statistical significance.

For girls in the normal-weight group, vital capacity demonstrated a significant negative correlation with the 50-m sprint (*r* = −0.230) and a significant positive correlation with the standing long jump (*r* = 0.252), while correlations with the 800-m run (*r* = −0.110) and sit-ups (*r* = 0.113) were not significant. Within the underweight, overweight, and obese female subgroups, following the rigorous Bonferroni correction, the correlation coefficients between vital capacity and all physical fitness test items—including the 50-m sprint, standing long jump, 800-m run, and sit-ups—failed to reach statistical significance.

### 3.3 Linear regression analysis of the correlation between pulmonary capacity and athletic performance

To control for confounding factors and examine the interactive and non-linear impacts of BMI and vital capacity on physical fitness test performance, hierarchical linear regression models were constructed. Model 1 incorporated the mean-centered BMI (BMI_c) and mean-centered vital capacity (Vital Capacity_c) as the main effect variables. Based on Model 1, Model 2 introduced the quadratic term of BMI (BMI_c^2^) to test for potential non-linear relationships. Model 3 further incorporated the first-order linear interaction term (Vital Capacity_c * BMI_c) and the second-order quadratic interaction term (Vital Capacity_c * BMI_c^2^), utilizing the Bootstrap method (1,000 resamples) to estimate the robust 95% confidence intervals (95% CIs) for the interaction terms.

The regression results for the boys’ group indicated that in Models 1 and 2 for the 50-m sprint, both BMI_c and Vital Capacity_c exhibited significant main effects (*p* < 0.005). In Model 3, although the conventional parametric test for the BMI_c^2^ term yielded a marginally significant *p*-value (*p* = 0.065), its robust 95% percentile confidence interval based on 1,000 Bootstrap resamples [(0.002, 0.026)] did not encompass zero. This finding suggests a potential non-linear trend in the moderating role of BMI on the 50-m sprint, which warrants further verification in larger sample sizes. For the 1,000-m run, the main effects of BMI_c and Vital Capacity_c were highly significant (*p* < 0.005); concurrently, the BMI_c^2^ term reached statistical significance (*β* = 0.340, *p* = 0.002), whereas neither the linear nor higher-order interaction terms attained significance. Regarding the standing long jump and pull-up items, Model 1 demonstrated that both BMI_c and Vital Capacity_c possessed significant main effects (*p* < 0.001), but the subsequently entered squared and interaction terms failed to reach statistical significance (*p* > 0.05; Table 4).

**Table 4:** Hierarchical regression analysis of BMI and vital capacity for boys.

Dependent Variable	Independent Variables / Interaction Terms	Model 1				Model 2				Model 3			
		*β*	*t*	*p*	(95% CI)	*β*	*t*	*p*	(95% CI)	*β*	*t*	*p*	(95% CI)
50-m sprint	BMI_c	0.221	3.615	0.003	(0.021, 0.094)	0.217	3.485	0.006	(0.180, 0.950)	0.147	1.979	0.075	(0.000, 0.084)
	Vital Capacity_c	−0.323	−5.277	<0.001	(0.000, 0.000)	−0.322	−5.246	<0.001	(0.000, 0.000)	−0.287	−3.855	<0.001	(0.000, 0.000)
	Vital Capacity_c × BMI_c					0.026	0.436	0.674	(−4.02 × 10^−5^, 5.13 × 10^−5^)	−0.021	−0.312	0.752	(−6.16 × 10^−5^, 3.56 × 10^−5^)
	BMI_c^2^									0.201	2.871	0.065	(0.020, 0.025)
	Vital Capacity_c × BMI_c^2^									−0.053	−0.598	0.606	(−1.97 × 10^−5^, 7.45 × 10^−6^)
Standing long jump	BMI_c	−0.347	−5.977	<0.001	(−3.270, −1.583)	−0.343	−5.818	<0.001	(−3.262, −1.533)	−0.318	−4.452	<0.001	(−3.154, −1.282)
	Vital Capacity_c	0.413	7.120	<0.001	(0.008, 0.016)	0.412	7.086	<0.001	(0.008, 0.016)	0.389	5.450	<0.001	(0.007, 0.016)
	Vital Capacity_c × BMI_c					−0.021	−0.382	0.696	(−0.001, 0.001)	−0.006	−0.089	0.933	(−0.001, 0.001)
	BMI_c^2^									−0.087	−1.304	0.327	(−0.370, 0.071)
	Vital Capacity_c × BMI_c^2^									0.039	0.459	0.693	(0.000, 0.000)
1,000-m run	BMI_c	0.322	5.304	<0.001	(1.746, 5.036)	0.305	4.966	<0.001	(1.433, 4.965)	0.172	2.423	<0.001	(0.254, 3.656)
	Vital Capacity_c	−0.280	−4.614	<0.001	(−0.019, −0.007)	−0.276	−4.556	<0.001	(−0.019, −0.006)	−0.238	−3.352	0.044	(−0.016, −0.005)
	Vital Capacity_c × BMI_c					0.097	1.670	0.233	(−0.001, 0.004)	0.003	0.043	0.979	(−0.003, 0.003)
	BMI_c^2^									0.340	5.090	0.002	(0.343, 1.211)
	Vital Capacity_c × BMI_c^2^									−0.044	−0.523	0.634	(−0.001, 0.000)
Pull-ups	BMI_c	−0.261	−4.211	<0.001	(−0.778, −0.305)	−0.251	−3.984	<0.001	(−0.764, −0.298)	−0.301	−3.963	0.002	(−1.027, −0.286)
	Vital Capacity_c	0.234	3.768	<0.001	(0.001, 0.003)	0.231	3.723	<0.001	(0.001, 0.003)	0.157	2.063	0.014	(0.000, 0.002)
	Vital Capacity_c × BMI_c					−0.059	−0.995	0.253	(0.000, 8.44 × 10^−5^)	−0.110	−1.559	0.094	(−0.001, 5.15 × 10^−5^)
	BMI_c^2^									0.005	0.066	0.946	(−0.061, 0.052)
	Vital Capacity_c × BMI_c^2^									0.153	1.689	0.062	(2.86 × 10^−6^, 0.000)

BMI_c = mean-centered body mass index; Vital Capacity_c = mean-centered vital capacity; β represents the standardized regression coefficient, and the p-value is calculated based on the Bootstrap standard error (after 1,000 sampling iterations). The values within parentheses represent the 95% Bootstrap confidence interval. Statistical inference is based on whether the confidence interval includes 0. Interaction Term is (Vital Capacity_c × BMI_c); Non-linear Interaction Term is ( $\text{Vital Capacity}\_c\times \mathrm{BMI}\_c^{2}$ ).

The regression analysis for the girls’ group revealed distinctly different characteristics compared to the boys’ cohort. For the 50-m sprint, BMI_c maintained a significant positive predictive effect in Models 1 and 2 (*p* < 0.05), yet its main effect faded to non-significance in Model 3 upon the inclusion of the interaction terms. All higher-order terms and interaction terms in Model 3 for the 50-m sprint lacked statistical significance (*p* > 0.05). For the 800-m run, only BMI_c demonstrated a highly significant main effect (Model 3: *β* = 0.391, *p* < 0.001), while Vital Capacity_c and its corresponding interaction terms were completely non-significant. In the standing long jump, the main effect of Vital Capacity_c remained continuously significant (Model 3: *β* = 0.221, *p* = 0.014); the main effect of BMI_c, although non-significant in Model 1 (*β* = −0.112, *p* = 0.102), shifted to statistical significance in Model 3 (*β* = −0.241, *p* = 0.012). Crucially, the first-order linear interaction term “Vital Capacity_c * BMI_c” attained statistical significance in this model (*β* = 0.192, *p* = 0.037). Additionally, for the one-minute sit-up item, the predictive effects of all entered variables were non-significant (*p* > 0.05; Table 5).

**Table 5:** Hierarchical regression analysis of BMI and vital capacity for girls.

Dependent variable	Independent variables/interaction terms	Model 1				Model 2				Model 3			
		*β*	*t*	*p*	(95% CI)	*β*	*t*	*p*	(95% CI)	*β*	*t*	*p*	(95% CI)
50-m sprint	BMI_c	0.190	2.885	0.002	(0.014, 0.066)	0.198	2.978	0.004	(0.014, 0.068)	0.263	3.092	0.005	(0.018, 0.091)
	Vital Capacity_c	−0.176	−2.671	0.015	(0.000, −5.50 × 10^−5^)	−0.177	−2.690	0.015	(0.000, −5.90 × 10^−5^)	−0.151	−1.912	0.064	(0.000, −1.77 × 10^−5^)
	Vital Capacity_c × BMI_c					0.060	0.908	0.390	(−5.50 × 10^−5^, 0.000)	0.093	1.319	0.207	(−3.50 × 10^−5^, 0.000)
	BMI_c^2^									−0.075	−0.919	0.321	(−0.011, 0.003)
	Vital Capacity_c × BMI_c^2^									−0.075	−0.898	0.393	(−3.10 × 10^−5^, 1.90 × 10^−5^)
Standing long jump	BMI_c	−0.112	−1.697	0.102	(−1.408, 0.065)	−0.118	−1.765	0.088	(−1.441, 0.032)	−0.241	−2.839	0.012	(−2.330, −0.378)
	Vital Capacity_c	0.207	3.126	0.005	(0.002, 0.010)	0.208	3.135	0.005	(0.002, 0.010)	0.221	2.799	0.014	(0.002, 0.012)
	Vital Capacity_c × BMI_c					−0.043	−0.647	0.477	(−0.003, 0.001)	−0.095	−1.347	0.167	(0.019, 0.434)
	BMI_c^2^									0.192	2.354	0.037	(−0.004, 0.001)
	Vital Capacity_c × BMI_c^2^									0.033	0.391	0.663	(0.000, 0.001)
800-m run	BMI_c	0.425	6.898	<0.001	(−0.428, 0.427)	0.423	6.801	<0.001	(−0.443, 0.387)	0.391	4.892	b	(−0.793, 0.316)
	Vital Capacity_c	−0.092	−1.501	0.151	(−0.001, 0.004)	−0.092	−1.494	0.159	(−0.001, 0.004)	−0.100	−1.343	0.224	(0.000, 0.004)
	Vital Capacity_c × BMI_c					−0.010	−0.166	0.874	(−0.001, 0.001)	−0.026	−0.393	0.757	(−0.002, 0.001)
	BMI_c^2^									0.042	0.549	0.671	(−0.022, 0.210)
	Vital Capacity_c × BMI_c^2^									0.028	0.360	0.794	(0.000, 0.000)
Sit-ups	BMI_c	−0.002	−0.024	0.985	(1.681, 3.373)	−0.008	−0.116	0.914	(1.649, 3.347)	−0.086	−0.985	0.359	(1.248, 3.349)
	Vital Capacity_c	0.079	1.172	0.255	(−0.007, 0.001)	0.080	1.186	0.244	(−0.007, 0.001)	0.124	1.526	0.102	(−0.009, 0.002)
	Vital Capacity_c × BMI_c					−0.048	−0.705	0.524	(−0.002, 0.002)	−0.074	−1.028	0.401	(−0.175, 0.304)
	BMI_c^2^									0.150	1.793	0.125	(−0.003, 0.002)
	Vital Capacity_c × BMI_c^2^									−0.041	−0.481	0.600	(−0.001, 0.001)

BMI_c = mean-centered body mass index; Vital Capacity_c = mean-centered vital capacity; β represents the standardized regression coefficient, and the *p*-value is calculated based on the Bootstrap standard error (after 1,000 sampling iterations). The values within parentheses represent the 95% Bootstrap confidence interval. Statistical inference is based on whether the confidence interval includes 0. Interaction Term is (Vital Capacity_c × BMI_c); Non-linear Interaction Term is ( $\text{Vital Capacity}\_c\times \mathrm{BMI}\_c^{2}$ ).

In summary, the hierarchical regression analysis across the continuous spectrum of the full sample demonstrated that the quadratic interaction terms (Vital Capacity_c * BMI_c^2^), which reflect continuous non-linear moderation, failed to reach statistical significance in the majority of the models (*p* > 0.05). Nevertheless, the Bootstrap 95% confidence intervals unveiled two noteworthy exceptions: the BMI_c^2^ term in the boys’ 50-m sprint and the Vital Capacity_c * BMI_c interaction term in the girls’ standing long jump. Although their conventional *p*-values might exceed 0.05 in certain contexts, their confidence intervals did not encompass zero, indicating that the moderating effect of BMI is not entirely absent. This statistical evidence demonstrates that instead of a global, continuous interaction effect across the entire sample spectrum, the moderation of BMI operates in a gender-specific and sports-item-selective pattern. Specifically, within the normal-weight range, the transmission of lung function into peripheral physical performance remains relatively stable; however, once the participants enter the overweight or obese physiological intervals, the association chain tends to exhibit structural heterogeneous fluctuations. Consequently, the discrepancies observed among the subgroups can only serve as exploratory evidence, suggesting a potential BMI-stratified association pattern. Given the cross-sectional design of this study and the absence of direct measurements regarding deep biological mechanisms, this stratified association feature and the potential trend of decoupling warrant further validation in future large-scale prospective cohort studies.

## 4 Discussion

Drawing on physical fitness test data from 493 eighth-grade students in a Beijing middle school, this study examined the associations between vital capacity and athletic performance across various body mass index (BMI) categories, while accounting for gender differences. The moderating effect of BMI was rigorously evaluated using bootstrapping-based confidence intervals. Results indicate that the relationship between vital capacity and physical fitness is characterized by gender- and BMI-stratified specificity. These findings provide novel exploratory evidence for understanding the mechanisms underlying individual differences in adolescent physical fitness and offer a preliminary quantitative basis for personalized interventions in physical education.

**Figure 1:**
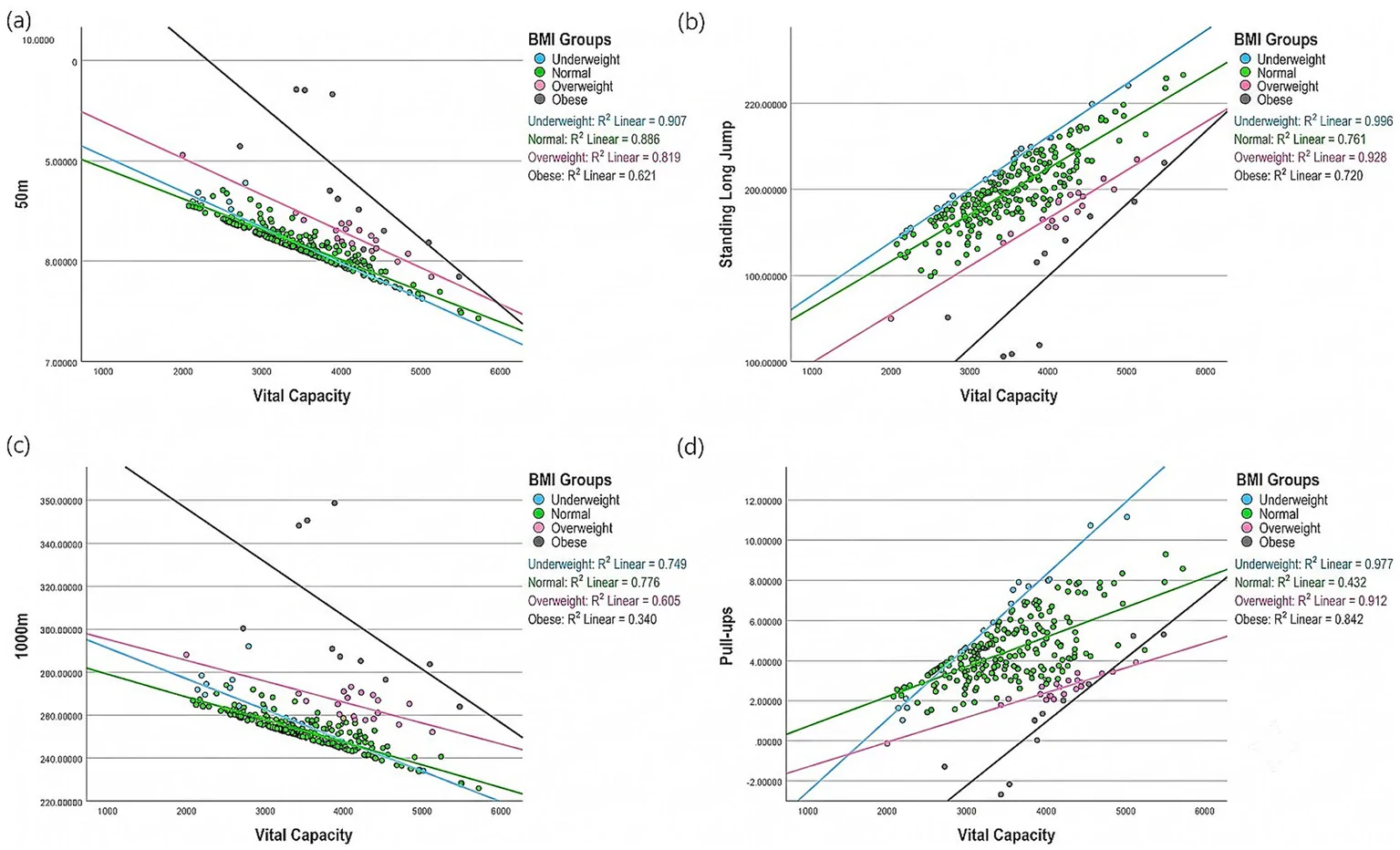
The Y-axis represents the non-standardized predicted values for each item, and the X-axis represents vital capacity. Subgroup color coding: Underweight (blue), Normal weight (green), Overweight (pink), and Obese (gray). **(a)** 50-m sprint, **(b)** 1000-m run, **(c)** Standing long jump, and **(d)** Pull-ups.

The predictive utility of vital capacity for athletic performance in underweight boys is limited, as evidenced by the lack of statistically significant correlations with the 50-meter sprint, standing long jump, 1,000-meter run, or pull-ups (*p* > 0.05). Figure 1 shows that the regression slopes are steepest for the standing long jump (*r*^2^ = 0.996) and pull-ups (*r*^2^ = 0.977), indicating a strong potential association between vital capacity and these specific performance metrics. However, descriptive analysis reveals that the average scores of underweight boys in the standing long jump and pull-ups remain lower than those of their normal-weight peers. From an exercise physiology perspective, both pull-ups and the standing long jump demand high levels of muscular power. Although underweight boys may possess a relative strength advantage, their limited skeletal muscle mass and insufficient glycogen stores may limit the ability to generate adequate contractile tension (15). Consequently, training strategies relying solely on cardiopulmonary loading may have limited efficacy for improving physical fitness in this demographic. Previous research has underscored a synergistic relationship between muscular strength and cardiorespiratory endurance in adolescent males, with strength training significantly enhancing movement efficiency (16). Therefore, it is recommended that underweight boys maintain standard comprehensive fitness training while incorporating a higher proportion of resistance-based strength exercises.

Participants in the normal-weight BMI group exhibited superior athletic performance across all tested categories, characterized by the lowest mean times for the 50-meter and 1,000-meter runs, the greatest mean distance for the standing long jump, and the highest mean frequency of pull-ups. Hierarchical regression analysis indicated that the main effect of mean-centered vital capacity was significant for all performance indicators (*p* < 0.005). Furthermore, in models incorporating interaction terms, the main effects for running events remained robust (50 m: *β* = −0.287, *p* < 0.001; 1,000 m: *β* = −0.238, *p* = 0.003), suggesting that vital capacity yields the greatest net benefit to performance in individuals with a normal BMI. As shown in Figure 1, the normal-weight BMI group displayed moderate negative regression slopes for running events and a moderate positive slope for the standing long jump. From a biomechanical perspective, a BMI within the standard range (18.5–23.9 kg/m^2^) is associated with an optimal proportion of fat-free mass, which minimizes the mechanical restriction of adipose tissue on diaphragmatic descent and thoracic expansion (17), allowing vital capacity to accurately reflect alveolar ventilation and diffusion capacity. Concurrently, adequate skeletal muscle mass acts as a ‘peripheral engine,’ facilitating efficient oxygen uptake and utilization, thereby enabling tight coupling between central ventilation and peripheral metabolism. Large-scale studies have further corroborated that students with a normal BMI exhibit optimal physical fitness, identifying a healthy weight status as the ‘golden window’ for maximizing the positive effects of vital capacity (18). Analysis of the overweight group demonstrates that the regression lines for the four performance indicators are essentially parallel to those of the normal-weight group, yet with higher intercepts; this indicates that at a given level of vital capacity, overweight boys exhibit poorer predicted performance. Running, as a cyclic locomotor activity, requires each stride to counteract gravity to propel the body forward and upward. The adipose tissue accumulated in overweight individuals functions as ‘metabolic dead weight,’ significantly increasing the mass-specific oxygen cost. Consequently, the limited oxygen supply derived from vital capacity is largely consumed by the heavy physical load, leaving insufficient capacity to support the additional oxygen requirements for muscle contraction. Although the static vital capacity of overweight boys (4119.85 mL) is higher than that of the normal-weight group, this ‘apparent advantage’ is significantly attenuated or negated during dynamic running. These findings are highly consistent with the limiting factor theory: when body composition deviates from the ideal range, the compensatory demand on the cardiovascular system to manage additional body mass rises sharply, significantly amplifying individual differences in vital capacity—a potential factor limiting speed, explosive power, and endurance performance (19). Existing research has also indicated that overweight adolescents exhibit reduced ventilatory efficiency and increased work of breathing during exercise, with relative VO_2_ max values significantly lower than those of their normal-weight peers (20). Therefore, for this population, improving cardiorespiratory function in conjunction with weight and fat reduction may represent a direct and effective pathway to unlocking their athletic potential.

The data from the obese group indicate that extreme body composition may have weakened the positive transformation efficiency between cardiopulmonary function and exercise performance, presenting a potential disconnection trend. Although this group exhibited the relatively high absolute vital capacity (4066.70 ± 808.26 mL), their athletic performance was the poorest across all categories. Correlation analysis revealed no significant association between vital capacity and performance in any of the tasks (*p* > 0.05), with several correlation coefficients showing an inverse direction compared to the normal-weight group. Scatter plots further reveal this disassociation trend, where the regression line for the 50-meter sprint is the steepest—suggesting that speed improves most noticeably with increasing vital capacity—yet the obese group’s 50-meter sprint performance remains inferior to that of the normal BMI group. Data points for the standing long jump and pull-ups were widely dispersed below the regression line of the normal-weight group, showing no trend of clustering as vital capacity improved. This phenomenon suggests that extreme body composition may remodel the link between cardiorespiratory function and physical performance. In obese individuals, factors such as intrathoracic fat accumulation and relative respiratory muscle insufficiency may result in high absolute vital capacity being accompanied by excessive body mass, which likely correlates with higher energy expenditure during physical activity, thereby potentially constraining dynamic physical performance (21). Consequently, as a practical implication derived from these exploratory findings, physical education interventions for obese students might adopt a ‘weight-management-first’ strategy, prioritizing the reduction of body fat and the improvement of body composition, while emphasizing low-impact, non-weight-bearing exercises to mitigate joint stress and injury risks.

**Figure 2:**
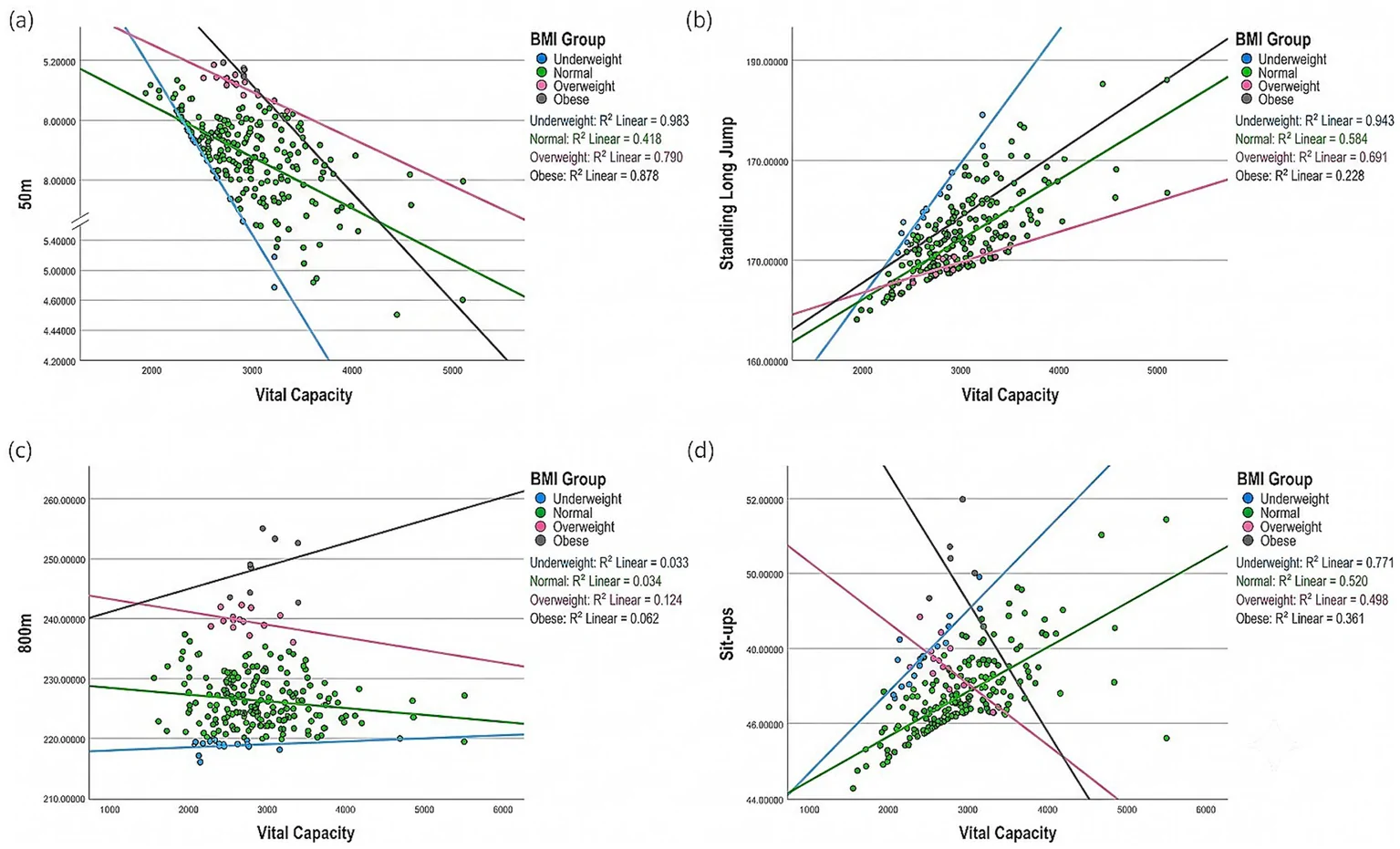
Scatter plots illustrating the slopes derived from stratified regression analysis for girls. The Y-axis represents the non-standardized predicted values for each item, and the X-axis represents vital capacity. Subgroup color coding: Underweight (blue), Normal weight (green), Overweight (pink), and Obese (gray). **(a)** 50-m sprint, **(b)** 800-m run, **(c)** Standing long jump, and **(d)** Sit-ups.

The analysis of the female group reveals a different regulatory pattern from that of the male group. Figure 2 demonstrates that for girls within the normal-weight range, performance in the 50-meter sprint and 800-meter run improves as vital capacity increases. Furthermore, the regression line for the standing long jump exhibits the steepest positive slope among the four tasks, with data points tightly clustered along the line, corroborating the significant BMI-squared term identified in the regression model (*β* = 0.192, *p* = 0.037). Within this standard BMI range, metabolic interference from body composition on cardiorespiratory energy supply is minimized, facilitating the efficient conversion of vital capacity into lower-limb explosive power. Consequently, in physical education practice, combined aerobic interval and lower-limb explosive power training can be designed to leverage this efficient conversion pathway for enhancing overall physical fitness.

However, the 800-meter run exhibits an opposite trend in overweight girls; performance is inferior to the normal-weight group, with non-significant interaction terms between vital capacity and BMI. The upward-shifted intercept in the scatter plot suggests that running speed is generally lower at any given level of vital capacity. During running, excess adipose tissue increases the mass-specific oxygen cost, consuming the oxygen supply derived from vital capacity to support the additional body load (17). Once obesity is reached, no significant correlation exists between vital capacity and athletic performance, and some regression lines even show a reversed direction, indicating a ‘decoupling’ of cardiorespiratory function from athletic performance. Although females possess a higher proportion of subcutaneous fat—which imposes less mechanical restriction on respiration—and estrogen promotes lipid oxidation to sustain metabolic efficiency to some degree, excessive fat accumulation in the obese state eventually exceeds the compensatory threshold, leading to a severe deterioration in dynamic ventilatory efficiency. Therefore, physical education interventions for obese students might prioritize weight management through fat reduction and body composition improvement, while emphasizing low-impact, non-weight-bearing exercises to mitigate joint stress and prevent excessive body fat from negating the potential benefits of cardiorespiratory function on endurance performance. For underweight girls, correlations between vital capacity and the 50-meter sprint, standing long jump, 800-meter run, and sit-ups were all non-significant (*p* > 0.05), and hierarchical regression revealed no interaction effects. The 800-meter regression line is the flattest, with performance times slightly increasing alongside vital capacity, while other tasks, despite having the steepest slopes among the four groups, lack predictive stability. Underweight girls with larger vital capacities often possess larger skeletal frames but insufficient muscle mass. Endurance performance depends on peripheral skeletal muscle oxygen utilization; thus, muscle deficiency limits peripheral oxygen uptake and reduces running economy. This physiological imbalance may prevent ventilatory advantages from being translated into athletic performance and may even hinder performance due to the additional energy expenditure required for trunk stabilization. Consequently, underweight girls should also undertake resistance training to increase lean body mass and enhance oxygen utilization capacity.

It is noteworthy that the hierarchical regression analysis conducted on the full sample failed to detect a robust global quadratic interaction term. However, this does not imply the absence of a moderating effect of BMI. Conversely, integrating the stratified regression slopes presented in Figures 1, 2, we observe that the moderating effect of BMI does not manifest as a smooth parabolic trend across BMI values; rather, it exhibits distinct, weight-stratified specificity: within the normal-weight range, the pathway through which vital capacity contributes to athletic performance remains unobstructed, whereas in the overweight and obese intervals, this pathway appears to be substantially attenuated. These findings suggest that the modulation of the association between vital capacity and athletic performance by BMI is more consistent with a ‘threshold effect’ rather than the traditional ‘curve effect’.

In the current landscape of school physical education, implementing fully individualized ‘one-on-one’ interventions remains challenging due to constraints in facilities and faculty-to-student ratios. The innovation of this study lies in transcending traditional empiricism—often summarized as ‘weight loss for the obese and muscle gain for the lean’—by quantifying the ‘boundary conditions’ under which vital capacity exerts its effects across distinct BMI phenotypes. Consequently, differentiated stratification is an essential pathway toward precise intervention. Differentiated grouping models—such as the ‘subject-based’ physical education currently piloted in domestic schools—have already yielded promising results (22).

Building on this, schools can implement ‘classroom micro-stratification’ by grouping students based on BMI screenings and designing tailored training tasks: comprehensive training emphasizing both aerobic capacity and explosive power for normal-weight students; weight-loss-focused aerobic training combined with strength exercises for overweight students; low-impact, non-weight-bearing exercises for obese students; and resistance training to increase lean mass for underweight students. Notably, the study reveals an apparent paradox: while obese boys exhibit the lowest athletic performance, they possess the highest mean absolute vital capacity. Biomechanically and physiologically, a larger overall body size and greater thoracic volume inherently facilitate higher absolute ventilation. However, this static advantage is eclipsed by the substantial mechanical and metabolic costs imposed by non-functional adipose tissue when these students must overcome gravity or their own body mass. This cautions educators against relying on absolute vital capacity as a misleading indicator when assessing the physical health of obese adolescents.

Crucially, implementing these interventions requires addressing the social-psychological risk: publicly grouping adolescents—who are highly sensitive to body image—by BMI can easily trigger body-related anxiety and stigma. To mitigate this, we propose ‘concealed micro-stratification’ rather than explicit, ‘labeling-based’ grouping. For instance, teachers can design unified circuit training routes where students perform individualized exercise intensities or movement variations at specific stations (e.g., explosive power or resistance training stations). This approach allows students to receive customized interventions aligned with their specific BMI-related requirements within a seemingly uniform classroom environment, thereby balancing scientific rigor with the protection of adolescent psychological dignity and motivation.

## 5 Conclusion

The moderating effect of BMI on the association between vital capacity and athletic performance in middle school students exhibits gender- and BMI-stratified specificity. The stratified subgroup analysis provides exploratory evidence regarding the relationship between lung function and physical fitness in middle school students, suggesting the existence of association patterns stratified by BMI. Given the limitations in sample size for extreme body composition subgroups and the fact that the underlying physiological mechanisms were not directly measured, the heterogeneous patterns revealed in this study should be interpreted with caution as preliminary exploratory findings. Nevertheless, this evidence suggests that a ‘one-size-fits-all’ approach to physical fitness interventions should be avoided in school physical education. This study puts forward a hypothesis for implicit stratified teaching strategies that integrate BMI and gender dimensions, which warrants further longitudinal validation and confirmation in large-scale cohort studies to provide more robust empirical support for precise interventions in adolescent physical health.

### 5.1 Limitations

This study is subject to several limitations. First, as daily physical activity was not included as a covariate, the strength of the direct association between vital capacity and athletic performance may have been partially overestimated; future studies should incorporate relevant questionnaires or objective monitoring devices to isolate these effects. Second, the sample sizes for extreme body composition subgroups, such as the obese boys, were relatively small (e.g., the post-hoc power for this subgroup was only 18.6%). Consequently, the findings and the derived stratified teaching strategies should be interpreted with caution as exploratory hypotheses, as they are not sufficient to support definitive causal inferences. Third, the cross-sectional design did not account for biological maturity; the phenomenon of ‘pulmonary developmental asynchrony’—which is common during puberty—may confound the results. Future research should prioritize larger sample sizes and incorporate maturity offset and multi-dimensional cardiorespiratory and body composition indices in longitudinal studies to validate these findings.

## Data Availability

The original contributions presented in the study are included in the article/supplementary material, further inquiries can be directed to the corresponding author/s.
